# Work and home productivity of people living with HIV in Zambia and South Africa

**DOI:** 10.1097/QAD.0000000000002160

**Published:** 2019-04-04

**Authors:** Ranjeeta Thomas, Rocco Friebel, Kerrie Barker, Lawrence Mwenge, Sarah Kanema, Nosivuyile Vanqa, Abigail Harper, Nomtha Bell-Mandla, Peter C. Smith, Sian Floyd, Peter Bock, Helen Ayles, Sarah Fidler, Richard Hayes, Katharina Hauck

**Affiliations:** a Department of Health Policy, London School of Economics and Political Science; b Faculty of Medicine, Imperial College London, London, UK; c ZAMBART Project, Ridgeway Campus, University of Zambia, Lusaka, Zambia; d Desmond Tutu TB Centre, Stellenbosch University, Department of Paediatrics and Child Health, Tygerberg Campus, Cape Town; e Division of Epidemiology and Biostatistics, University of the Witwatersrand, Johannesburg, South Africa; f Business School, Imperial College London; g Department of Infectious Disease Epidemiology, Faculty of Epidemiology and Population Health; h Department of Clinical Research, Faculty of Infectious and Tropical Diseases, London School of Hygiene and Tropical Medicine; i Department of Medicine; j Department of Infectious Disease Epidemiology, Imperial College London, London, UK.

**Keywords:** absenteeism, economics, HIV/AIDS, informal sector, labour productivity, sickness days

## Abstract

**Objective:**

To compare number of days lost to illness or accessing healthcare for HIV-positive and HIV-negative individuals working in the informal and formal sectors in South Africa and Zambia.

**Design:**

As part of the HPTN 071 (PopART) study, data on adults aged 18–44 years were gathered from cross-sectional surveys of random general population samples in 21 communities in Zambia and South Africa. Data on the number of productive days lost in the last 3 months, laboratory-confirmed HIV status, labour force status, age, ethnicity, education, and recreational drug use was collected.

**Methods:**

Differences in productive days lost between HIV-negative and HIV-positive individuals (’excess productive days lost’) were estimated with negative binomial models, and results disaggregated for HIV-positive individuals after various durations on antiretroviral treatment (ART).

**Results:**

From samples of 19 330 respondents in Zambia and 18 004 respondents in South Africa, HIV-positive individuals lost more productive days to illness than HIV-negative individuals in both countries. HIV-positive individuals in Zambia lost 0.74 excess productive days [95% confidence interval (CI) 0.48–1.01; *P* < 0.001] to illness over a 3-month period. HIV-positive in South Africa lost 0.13 excess days (95% CI 0.04–0.23; *P* = 0.007). In Zambia, those on ART for less than 1 year lost most days, and those not on ART lost fewest days. In South Africa, results disaggregated by treatment duration were not statistically significant.

**Conclusion:**

There is a loss of work and home productivity associated with HIV, but it is lower than existing estimates for HIV-positive formal sector workers. The findings support policy makers in building an accurate investment case for HIV interventions.

## Abstract 2

Supplemental Digital Content is available in the text

## Introduction

The majority of people living with HIV (PLWH) are in their most productive phase of life. Worldwide, 78% of individuals living with HIV are between the ages of 15 and 49 [1], and most of them are either working, studying or engaged in housework and caring for children or the elderly. Prior to the expansion of antiretroviral therapy (ART) in low-income countries, health status and productivity of HIV-positive individuals declined as HIV infection progressed to AIDS and premature death. This had serious consequences for the social and economic situations of PLWH and their households. The expanded availability of ART rapidly restored health and physical functioning [2] and extended life expectancy [3,4], thereby restoring and maintaining worker productivity and the well being of households [5,6].

The success of ART in safe-guarding the livelihoods of PLWH and their households plays an important role in motivating the global response to the epidemic [7]. The ambitious 2015 UNAIDS Fast Track Targets for improved access to treatment are partly motivated by modelling predictions of large economic gains because of improved labour productivity [8,9]. These predictions are based on evidence of how the productivity of workers in formal employment recovers before and after ART initiation. It is unclear, however, whether the predictions apply to the wider HIV-infected population. In low-income and middle-income countries (LMICs), the majority of individuals are not formally employed; in sub-Saharan Africa, informal sector workers make up 88% of the labour force [10]. They often have precarious informal employment without contracts, no paid sick leave, lower wages and longer working hours, and therefore, face different incentives with respect to absenteeism than formal sector workers. Informal workers may simply be unable to afford taking days off work. Individuals engaged in housework and those studying make valuable contributions to households, communities and the economy, but have also been excluded from most previous analyses.

We conducted this study in two of the most HIV/AIDS-affected countries globally; South Africa and Zambia, to compare the productive days lost (PDLs) by PLWH with those lost by HIV-negative individuals. It is the first analysis of the association between PDLs and HIV/AIDS in a random sample of adults. It includes individuals in formal and informal employment, and those not in the labour force. Most previous studies that estimated excess PDLs analysed employees at one or a few companies, including tea plantations in Kenya [11–14], mining companies in South Africa and Botswana [5,15] and a public sector organization in Zambia [16] (see A1 for a literature review). Studies were mostly small in scale, with a median sample size of 2051 (min: 87, max: 7666), which included a median number of HIV-positive of 237 (min: 11, max: 1703). Our study provides a rare insight into the productivity of PLWH at all stages of engagement with HIV care, including PLWH before diagnosis. Only three previous studies [15,17,18] analysed PLWH at all stages of disease, whereas four [5,12–14] analysed HIV-positive employees before and after initiation of ART, and two [11,16] focused exclusively on PLWH shortly before death. Our study design enabled adjustment for confounders that were collected for HIV-positive and HIV-negative in the same way. HIV status was determined from blood samples taken during the survey and confirmed with laboratory testing. All previous studies except three [15,17,18] benchmarked HIV-positive individuals against employees with unknown HIV status, therefore, the comparison groups used by previous studies may have been distorted by an unknown number of HIV-positive individuals.

## Methods

### Study population and data

The survey was conducted as the baseline of the on-going HPTN 071 (PopART) cluster-randomized trial measuring the effect of a combination prevention intervention on population level HIV-incidence [19]. HPTN 071 was implemented in 21 communities: 12 in Zambia covering four provinces and six districts, and nine in South Africa in the Cape Metro and Cape Winelands districts of the Western Cape Province (A2).

The study population is a cross-sectional random sample of adults between 18 and 44 years, residing within a household in the communities enrolled in the HPTN 071 (PopART) trial. Study participants consented to complete a research questionnaire, and to donate a venous blood sample annually, which was tested for HIV using a fourth-generation assay (A3). The data used in this article was gathered between November 2013 and March 2015.

From each randomly selected household, one adult was randomly selected for participation in the survey (A4). The survey gathered information on HIV testing, sociodemographics, health, economic and behavioural variables. PDLs were measured as responses to the question ‘*In the last 3 months, how many days have you been prevented from doing your usual work due to your own sickness or seeking healthcare?*’ We followed convention in the labour economics literature and defined ‘in the labour force’ (ILF) as those self-reporting being currently employed, self-employed, unemployed (looking for work or waiting to start new work) or waiting to continue agricultural work [20]. ‘Not in the labour force’ (NILF) included homemakers, students, retirees and others not looking for work. Those reporting being permanently sick or disabled were excluded from the analysis. If respondents self-reported being HIV-positive, information was gathered on whether they were in HIV care, and whether and for how long they had been on ART.

A full ethics review of the trial protocol (DOI https://doi.org/10.1186/1745-6215-15-57) was done by the ethics committees of the University of Zambia, University of Stellenbosch, and the London School of Hygiene and Tropical Medicine.

### Statistical analysis

We used multivariate negative binomial regression (NegBin) models with a quadratic variance function to evaluate the effect of HIV status on PDL. The NegBin model is appropriate as the dependent variable PDL is over-dispersed [21] with a variance greater than its mean (A5). We used STATA (version 14; STATA Corp., College Station, Texas, USA) and its *nbreg* routine for estimation, and *countfit* routine for evaluating model fit of the NegBin compared with a standard Poisson model [22,23].

Results are presented as both marginal effects and predicted values evaluated at the means of all other covariates. A positive marginal effect represents the additional or ‘excess PDLs’ that PLWH lose because of illness and/or accessing healthcare over 3 months when compared with HIV-negative individuals. The predicted value represents the total PDLs for specified subgroups of the sample, measured in number of days over 3 months. Two model specifications per country were estimated. In the first specification (models 1a and 2a), HIV status was classified as a binary indicator representing laboratory-confirmed HIV-positive and HIV-negative individuals. In the second specification (models 1b and 2b), four categories of HIV-positive status were defined, with HIV-negative individuals as the base case: HIV-positive and not on ART, HIV-positive on ART less than 1 year, HIV-positive on ART 1–2 years and HIV-positive on ART 3 or more years. The models included as adjustment variables: age, sex, education, ethnic group, use of recreational drugs and labour force participation status. All models also included dummy variables for each community to capture unobservable differences across communities. Models were estimated separately for Zambia and South Africa.

## Results

The full survey sample included responses from 19 750 (83%) of 23 676 randomly selected individuals in Zambia and 18 941 (88%) of 21 568 randomly selected individuals in South Africa (Table 1). Laboratory-confirmed HIV status was available for 19 330 (98%) participants in Zambia and 18 004 (95%) in South Africa; of whom 4128 (21%) and 4012 (22%) were HIV-positive, respectively. In both countries, the majority of PLWH reported not being on ART. Amongst those HIV-positive and on ART, the largest proportion were on ART for 3 years or more, followed by on ART for less than 1 year. The mean number of PDLs reported for the 3-month period before the interview was 1.3 days (SD: 6.11 days) for participants from Zambia and 0.31 days (SD: 3.0 days) for participants from South Africa. Among PLWH in Zambia, 13% reported having more than three PDLs in the past 3 months, compared with 7% of HIV-negative individuals. There was no difference between the two groups in South Africa (2%). In both countries, average PDLs were higher for HIV-positive (Zambia: 2.17, SD: 8.31; South Africa: 0.48, SD: 4.29) than HIV-negative (Zambia: 1.03, SD: 5.25; South Africa: 0.26, SD: 2.59) individuals. The majority of respondents were women and had completed secondary education. Labour force participation was higher in South Africa than in Zambia.

**Table 1:** Demographic and socioeconomic characteristics of a random sample of adults 18–44 years of age in 21 communities in Zambia and South Africa.

	Zambia		South Africa	
	*N* = 19 750		*N* = 18 941	
Productive days lost (PDLs) in the last 3 months	1.3	6.11	0.31	3
PDLs, HIV-positive	2.17	8.31	0.48	4.29
PDLs, HIV-negative	1.03	5.25	0.26	2.59
PDLS >3 days, HIV-positive	526/3952	13%	87/3821	2%
PDLS >3 days, HIV-negative	1069/14 496	7%	208/12 862	2%
Age under 25 years	8894/19 730	45%	6355/18 610	34%
Age 25–34 years	7193/19 730	37%	7597/18 610	41%
Age 35– 44 years	3643/19 730	18%	4658/18 610	25%
Sex				
Male	5428/19 733	28%	5816/18 612	31%
Female	14 305/19 733	72%	12 796/18 612	69%
Labour force participation				
In the labour force	8785/18 623	47%	15 133/18 400	82%
Not in the labour force	9799/18 623	53%	3112/18 400	17%
Unable to work (permanently sick or injured)	39/18 623	0%	155/18 400	1%
Ethnic group				
	Bemba 5827/19 750	30%	Xhosa 12 048/18 941	64%
	Tonga 2453/19 750	12%	Multiracial 4803/18 941	25%
	Lozi 1547/19 750	8%	Afrikaans 526/18 941	3%
	Chewa 1404/19 750	7%	Other^a^ 1564/18 941	8%
	Other^a^ 8519/19 750	43%		
Education level				
School education less than grade 8 (primary school)	5544/19 668	28%	1472/18 466	8%
School education between grades 8 and 12 (secondary school)	12 808/19 668	65%	15 947/18 466	86%
College, university, or other higher education	1316/19 668	7%	1047/18 466	6%
Use recreational drugs	480/19 629	2%	689/18 432	4%
HIV-status				
HIV-negative	15 202/19 330	79%	13 992/18 004	78%
HIV-positive	4128/19 330	21%	4012/18 004	22%
HIV-positive not on ART^b^^,^^c^	2446/4128	59%	2592/4012	65%
HIV-positive on ART <1 year^b^	509/4128	12%	351/4012	9%
HIV-positive on ART 1–2 years^b^	347/4128	8%	268/4012	7%
HIV-positive on ART 3 or more years^b^	714/4128	17%	574/4012	14%
Unknown ART status^b^^,^^d^	112/4128	3%	227/4012	6%

Data are mean (SD), *n* (%), or *n*/*N* (%).

^a^All other ethnic groups varied between 0.03 and 6.69%.

^b^ART status at the start of the 3-month recall period of PDLs; numbers based on responses by those self-reporting being HIV-positive.

^c^Includes respondents with lab confirmed HIV-positive status who did not self-report being HIV-positive.

^d^Includes respondents with missing self-reported ART status.

In Zambia, PLWH lost a total of 1.70 days over 3 months on average [95% confidence interval (CI) 1.44–1.95, Table 2]; these were 0.74 ‘excess PDLs,’ that is, 0.74 more days (95% CI 0.48–1.01; *P* < 0.001, model 1a, Table 3) than HIV-negative individuals, who lost a total of 0.95 (95% CI 0.88–1.02) days (Table 2). Compared with HIV-negative individuals, being on ART for less than 1 year was associated with the largest number of excess PDLs (1.24; 95% CI 0.34–2.14; *P* = 0.007, model 1b), followed by being on ART between 1 and 2 years (1.08; 95% CI 0.06–2.11; *P* = 0.038, model 1b), being on ART for three or more years (0.79; 95% CI 0.16–1.41; *P* = 0.014, model 1b) and being HIV-positive but not on ART (0.61; 95% CI 0.30–0.92; *P* < 0.001, model 1b). In South Africa, PLWH lost a total of 0.31 days over 3 months on average (95% CI 0.22–0.40), an excess of 0.13 days (95% CI 0.04–0.23; *P* = 0.007, model 2a) compared with HIV-negative individuals who lost 0.18 days in total (95% CI 0.15–0.20). We found no significant differences when HIV-status was disaggregated by duration on ART for individuals in the South African communities.

**Table 2:** Predicted productive days lost over 3 months, by HIV-status and for various subgroups.

		Zambia						South Africa				
Subgroups	HIV-negative^b^	HIV-positive^a^	HIV-positive not on ART^b^	HIV-positive on ART <1 year^b^	HIV-positive 1-2 years^b^	HIV-positive on ART 3 years +^b^	HIV-negative^b^	HIV-positive^a^	HIV-positive not on ART^b^	HIV-positive on ART < 1 year^b^	HIV-positive 1-2 years^b^	HIV-positive on ART 3 years +^b^
All	0.95 [0.876–1.023]	1.70 [1.441–1.952]	1.56 [1.261–1.858]	2.19 [1.296–3.084]	2.03 [1.011–3.056]	1.74 [1.119–2.357]	0.18 [0.149–0.201]	0.31 [0.220–0.400]	0.21 [0.136–0.280]	1.58 [0.172–2.997]	0.35 [−0.011 to 0.714]	0.18 [0.050–0.307]
Under 25 years	0.75 [0.675–0.834]	1.34 [1.093–1.590]	1.24 [0.972–1.506]	1.74 [1.006–2.475]	1.62 [0.780–2.453]	1.38 [0.860–1.902]	0.14 [0.105–0.168]	0.23 [0.143–0.319]	0.16 [0.095–0.227]	1.23 [0.083–2.380]	0.27 [−0.019 to 0.566]	0.14 [0.032–0.246]
25 - 34 years	1.01 [0.885–1.130]	1.80 [1.500–2.109]	1.65 [1.312–1.996]	2.32 [1.359–3.287]	2.16 [1.069–3.246]	1.84 [1.169–2.518]	0.18 [0.136–0.215]	0.32 [0.220–0.424]	0.21 [0.130–0.287]	1.59 [0.169–3.008]	0.35 [−0.008 to 0.713]	0.18 [0.046–0.311]
35–44 years	1.46 [1.212–1.717]	2.62 [2.124–3.122]	2.40 [1.846–2.963]	3.38 [1.941–4.812]	3.14 [1.520–4.751]	2.68 [1.717–3.641]	0.25 [0.177–0.317]	0.43 [0.282–0.587]	0.29 [0.173–0.413]	2.24 [0.195–4.282]	0.50 [−0.028 to 1.023]	0.25 [0.072–0.432]
Female	0.95 [0.864–1.035]	1.70 [1.438–1.957]	1.56 [1.257–1.862]	2.19 [1.293–3.086]	2.03 [1.014–3.052]	1.74 [1.119–2.355]	0.21 [0.169–0.242]	0.35 [0.246–0.458]	0.24 [0.158–0.330]	1.86 [0.183–3.541]	0.41 [−0.012 to 0.839]	0.21 [0.060–0.360]
Male	0.95 [0.825–1.076]	1.69 [1.363–2.024]	1.56 [1.209–1.912]	2.19 [1.260–3.124]	2.04 [0.971–3.099]	1.74 [1.077–2.401]	0.12 [0.092–0.153]	0.23 [0.148–0.317]	0.15 [0.085–0.205]	1.11 [0.108–2.107]	0.25 [−0.016 to 0.508]	0.12 [0.028–0.221]
School education < grade 8 (primary)	1.07 [0.926–1.223]	1.92 [1.577–2.262]	1.76 [1.384–2.145]	2.48 [1.440–3.517]	2.30 [1.120–3.483]	1.97 [1.249–2.684]	0.26 [0.136–0.385]	0.60 [0.282–0.927]	0.31 [0.123–0.495]	2.36 [0.154–4.561]	0.52 [−0.073 to 1.120]	0.27 [0.040–0.491]
School education grades 8–12 (secondary)	0.89 [0.805–0.967]	1.58 [1.326–1.839]	1.45 [1.165–1.745]	2.04 [1.199–2.887]	1.90 [0.939–2.855]	1.62 [1.033–2.209]	0.17 [0.146–0.202]	0.30 [0.212–0.389]	0.21 [0.135–0.277]	1.57 [0.159–2.986]	0.35 [−0.010 to 0.708]	0.18 [0.049–0.305]
College, university, or other higher education	1.10 [0.816–1.390]	1.97 [1.383–2.554]	1.81 [1.231–2.391]	2.54 [1.332–3.756]	2.36 [1.016–3.708]	2.02 [1.126–2.910]	0.11 [0.049–0.181]	0.20 [0.068–0.328]	0.14 [0.045–0.227]	1.04 [−0.066 to 2.143]	0.23 [−0.049 to 0.510]	0.12 [0.008–0.226]
In the labour force	1.05 [0.941–1.165]	1.88 [1.572–2.193]	1.73 [1.378–2.080]	2.43 [1.430–3.425]	2.25 [1.104–3.405]	1.93 [1.229–2.623]	0.18 [0.151–0.210]	0.32 [0.228–0.414]	0.21 [0.140–0.289]	1.64 [0.181–3.091]	0.36 [−0.011 to 0.738]	0.18 [0.052–0.316]
Not in the labour force	0.86 [0.776–0.951]	1.54 [1.285–1.798]	1.42 [1.130–1.707]	1.99 [1.162–2.822]	1.85 [0.916–2.783]	1.58 [1.005–2.155]	0.15 [0.100–0.197]	0.26 [0.140–0.372]	0.18 [0.091–0.261]	1.34 [0.053–2.634]	0.30 [−0.023 to 0.620]	0.15 [0.029–0.273]
Bemba (Zambia)/Xhosa (South Africa)	0.77 [0.673–0.875]	1.38 [1.122–1.648]	1.27 [0.986–1.556]	1.78 [1.032–2.537]	1.66 [0.803–2.511]	1.42 [0.884–1.948]	0.16 [0.128–0.187]	0.28 [0.199–0.359]	0.19 [0.122–0.251]	1.43 [0.155–2.696]	0.32 [−0.008 to 0.641]	0.16 [0.045–0.276]
Tonga (Zambia)/Multiracial (South Africa)	1.25 [1.008–1.496]	2.22 [1.694–2.750]	2.06 [1.511–2.600]	2.89 [1.588–4.186]	2.68 [1.231–4.131]	2.29 [1.381–3.199]	0.28 [0.207–0.344]	0.48 [0.282–0.676]	0.33 [0.183–0.471]	2.50 [0.157–4.834]	0.55 [−0.040 to 1.148]	0.28 [0.062–0.500]
Lozi (Zambia)/Afrikaans (South Africa)	1.20 [0.901–1.503]	2.16 [1.581–2.744]	1.97 [1.383–2.564]	2.77 [1.491–4.052]	2.57 [1.166–3.981]	2.20 [1.279–3.118]	0.72 [−0.383 to 1.823]	1.26 [−0.719 to 3.240]	0.85 [−0.475 to 2.181]	6.51 [−4.959 to 17.984]	1.45 [−1.212 to 4.104]	0.73 [−0.502 to 1.968]
Chewa (Zambia)	1.26 [0.930–1.580]	2.23 [1.587–2.882]	2.06 [1.418–2.703]	2.89 [1.509–4.280]	2.69 [1.174–4.202]	2.30 [1.301–3.292]						
Other	0.92 [0.820–1.026]	1.65 [1.371–1.923]	1.52 [1.207–1.824]	2.13 [1.243–3.015]	1.98 [0.975–2.979]	1.69 [1.072–2.305]	0.07 [0.032–0.112]	0.14 [0.055–0.216]	0.09 [0.032–0.140]	0.65 [−0.011 to 1.320]	0.15 [−0.027 to 0.318]	0.07 [0.009–0.139]

Confidence intervals of the mean prediction from a hypothesis test of significant difference from zero; PDLs for aggregate categories not displayed in the table can be approximated by the weighted averages of PDLs across the respective disaggregated categories. For example to PDLs for the 1570 (509 + 347 + 714) individuals on ART in Zambia can be calculated as (509^*^2.19  + 347^*^2.03 + 714^*^1.74)/1570 = 1.95; differences in predicted values may deviate slightly from marginal effects presented in Table 3 because of the nonlinearity of the models.

^a^Predictions are based on models 1a (Zambia) and 2a (South Africa), and generated at sample mean values of all other covariates.

^b^Predictions are based on models 1b (Zambia) and 2b (South Africa), and generated at sample mean values of all other covariates.

**Table 3:** Multivariable analysis of factors associated with productive days lost.

	Zambia		South Africa	
	Model 1a	Model 1b	Model 2a	Model 2b
HIV-negative (base)	--	--	--	--
				
HIV-positive	0.74^***^[0.48–1.01]		0.13^***^[0.04–0.23]	
	*P* < 0.001		*P* = 0.007	
HIV-positive not on ART		0.61^***^[0.30–0.92]		0.03[−0.05 to 0.11]
		*P* < 0.001		*P* = 0.416
HIV-positive on ART < 1 year		1.24^***^[0.34–2.14]		1.41 [−0.004 to 2.82]
		*P* = 0.007		*P* = 0.051
HIV-positive on ART 1–2years		1.08^**^[0.06–2.11]		0.18 [−0.19 to 0.54]
		*P* = 0.038		*P* = 0.341
HIV-positive on ART 3 or more years		0.79^**^[0.16–1.41]		0 [−0.13 to 0.14]
		*P* = 0.014		*P* = 0.961
In the labour force (base)	--	--	--	--
				
Not in the labour force	−0.05 [−0.21 to 0.11]	−0.04 [−0.20 to 0.12]	0 [−0.08 to 0.08]	0.001 [−0.07 to 0.07]
	*P* = 0.571	*P* = 0.592	*P* = 0.989	*P* = 0.982
Under 25 years (base)	--	--	--	--
				
25–34 years	0.25^***^ [0.08–0.41]	0.24^***^ [0.08–0.40]	0.06^**^ [0.01–0.12]	0.05 [−0.01 to 0.10]
	*P* = 0.003	*P* = 0.004	*P* = 0.031	*P* = 0.082
35–44 years	0.73^***^[0.44–1.02]	0.72^***^[0.43–1.02]	0.11^***^ [0.03–0.19]	0.11^***^[0.03–0.19]
	*P* < 0.001	*P* < 0.001	*P* = 0.006	*P* = 0.006
Female (base)	--	--	--	--
				
Male	−0.05 [−0.22 to 0.12]	−0.04 [−0.21 to 0.13]	−0.08^***^[−0.14 to −0.03]	−0.10^***^ [−0.15 to −0.05]
	*P* = 0.593	*P* = 0.626	*P* = 0.002	*P* = < 0.000
Bemba (base Zambia)/Xhosa (base South Africa)	--	--	--	--
				
Tonga (Zambia)/Multiracial (South Africa)	0.13 [−0.16 to 0.41]	0.14 [−0.15 to 0.42]	0.01 [−0.09 to 0.11]	0.01 [−0.08 to 0.10]
	*P* = 0.383	*P* = 0.353	*P* = 0.841	*P* = 0.850
Lozi (Zambia)/Afrikaans (South Africa)	0.15 [−0.20 to 0.49]	0.14 [−0.20 to 0.48]	0.87 [−0.84 to 2.59]	0.81 [−0.77 to 2.40]
	*P* = 0.403	*P* = 0.418	*P* = 0.319	*P* = 0.314
Chewa (Zambia)	0.22 [−0.12 to 0.57]	0.23		
	*P* = 0.209	[−0.12 to 0.57] *P* = 0.201		
Other	0.03 [−0.14 to 0.20]	0.03 [−0.14 to 0.21]	−0.06 [−0.14 to 0.02]	−0.07^**^ [−0.14 to −0.00]
	*P* = 0.728	*P* = 0.711	*P* = 0.164	*P* = 0.045
School education less than grade 8 (primary school, base)				
				
School education between grades 8 and 12 (secondary school)	−0.23^**^ [−0.43 to −0.03]	−0.23^**^ [−0.43 to −0.03]	−0.13 [−0.29 to 0.03]	−0.04 [−0.16 to 0.08]
	*P* = 0.026	*P* = 0.025	*P* = 0.228	*P* = 0.513
College, university, or other higher education	−0.2 [−0.54 to 0.14]	−0.19 [−0.53 to 0.15]	−0.14 [−0.33 to 0.04]	−0.05 [−0.20 to 0.10]
	*P* = 0.260	*P* = 0.265	*P* = 0.135	*P* = 0.505
Does not use recreational drugs				
				
Uses recreational drugs	0.54 [−0.16 to 1.24]	0.53 [−0.17 to 1.22]	0.14 [−0.09 to 0.36]	0.16 [−0.07 to 0.40]
	*P* = 0.131	*P* = 0.137	*P* = 0.235	*P* = 0.169
Community fixed effects	Yes	Yes	Yes	Yes
*N*	17 397	17 324	16 219	16 086
Likelihood ratio test (*H*_o_: *α* = 0)				
*χ*^2^	86 000	85 000	26 000	26 000
Prob at least *χ*^2^	0.0000	0.0000	0.0000	0.0000

Symbols (^***^) and (^**^) denote 99% and 95% statistical confidence levels, respectively; presented are marginal effects evaluated at the means of all other covariates; data are change in mean PDLs (95% CI), unless otherwise stated. For all factor variables, each category is compared with the base category. ART, antiretroviral therapy; CI, confidence interval.

When examining differences in total predicted values for both countries, PDLs were much lower in South Africa than in Zambia for nearly all subgroups (Table 2). Of the four groups formed based on HIV and labour force status, HIV-positive ILF had highest PDLs at 1.88 (95% CI 1.57–2.19) and 0.32 (95% CI 0.23–0.41) in Zambia and South Africa, respectively, followed by HIV-positive NILF at 1.54 (95% CI 1.28—1.80) and 0.26 (95% CI 0.14–0.37), HIV-negative ILF at 1.05 (95% CI 0.94–1.17) and 0.18 (95% CI 0.15–0.21), and HIV-negative NILF with lowest PDLs at 0.86 (95% CI 0.78–0.95) and 0.15 (95% CI 0.10–0.20). Predicted PDLs increased with age. Among HIV-positive individuals, 35–44-year olds who had been on ART for less than 1 year had highest PDLs at 3.38 days (95% CI 1.94–4.81) in Zambia and 2.24 days (95% CI 0.19–4.28) in South Africa, whereas under 25-year olds who were not on ART had lowest PDLs at 1.24 days (95% CI 0.97–1.51) in Zambia, and 0.16 days (95% CI 0.09–0.23) in South Africa.

There was no substantial sex difference in predicted PDLs among HIV-positive and HIV-negative individuals in both countries. HIV-negative and HIV-positive individuals across all categories who had completed secondary school had lower PDLs than those with primary school and higher education in Zambia. In South Africa, those with higher education had fewest PDLs, followed by those with secondary education and those with primary education. Predicted PDLs also differed across regions in Zambia**,** but there was little variation across regions in South Africa. Likelihood ratio tests for comparison with the Poisson model rejected the null of no over-dispersion (Table 3), confirming that NegBin provided a better fit (A6).

## Discussion

This is the first study of productive days lost to illness or accessing healthcare among HIV-positive and HIV-negative individuals in a random sample of adults in sub-Saharan Africa. It offers a rare insight into PDLs for the large majority of the population that is informally employed, self-employed, unemployed or not part of the labour force. The study further provides estimates of the PDLs of PLWH at different stages of engagement with HIV care, including those not on treatment, and those who were unaware of their status (44% in Zambia and 53% in South Africa) [2]. We undertook a direct comparison of PDLs between HIV-positive and HIV-negative individuals based on laboratory-confirmed HIV status. HIV-negative individuals provided an important benchmark that allowed us to analyse the association between HIV and PDLs, which is a crucial information in countries with competing risks that impede productivity, most notably other diseases. We analysed PDLs, which were lost to both sickness and accessing healthcare. Travel and waiting times at facilities have been identified as important barriers to accessing and remaining in HIV care [24]. We performed analyses separately for Zambia and South Africa because of substantial differences in labour markets, social security and healthcare systems.

In Zambia, 21% of the sample were HIV-positive and had 0.74 more PDLs than HIV-negative individuals over 3 months, whereas in South Africa, the 22% PLWHs had only 0.13 more PDLs. Our estimates are markedly lower than those from previous studies [5,11–17]; the median excess PDLs across eight previous studies was 5.1 days over 3 months, with high SD of 9.55 and estimates ranging between zero and over 33 excess PDLs for HIV-positive workers in their final year of life. Previous studies analysed PLWH in formal employment who were not representative of the population of PLWH, which may explain some of the divergence. Most formal sector workers enjoy statutory paid sick leave and have, therefore, lower opportunity costs of work absenteeism. Most respondents in our sample were informal sector workers, or unemployed workers with informal jobs and less able to afford a day of lost pay. This may explain why our estimates are lower than those of previous studies. Moreover, our disaggregated results for Zambia indicate that the two HIV-positive fractions with the lowest excess PDLs, that is, those not on ART and those on ART for 3 years or more together, make up 76% of the HIV-positive population. It seems reasonable that these groups lose fewer days than those more recently started on ART, because the former are in the earlier stages of the disease (and therefore, not yet on ART), and the latter are virally suppressed because they have been on ART long-term. Our comparison of community-level variations in excess PDLs showed significant differences within Zambia, but less so across communities in South Africa. These differences may be driven by a range of unobservable factors that are not captured in the model, including variations in economic conditions across regions, health system differences and social norms. The larger variations observed in Zambia are most likely because the study communities are spread across the country, reflecting the heterogeneity across regions, whereas in South Africa, the communities are all located in the Western Cape Province, and thus more likely to be similar in unobservable characteristics.

Five of nine previous studies were conducted before 2010 when ART was less accessible, or they focused on the (nowadays) small and nonrepresentative subgroup of PLWH in their final year of life, or with AIDS [11,15–17], and it is likely that they had higher PDLs than the population of PLWH today. Longitudinal studies among infected agricultural and mining workers are consistent with our findings. They have demonstrated a V-shaped pattern for labour force participation and productivity over the course of HIV disease, declining sharply as symptoms worsen in the months before ART initiation and rebounding within a few months to levels close to those experienced prior to becoming symptomatic [12,13,25–27]. Across all CD4^+^ cell count ranges except less than 50 μl, PLWH receiving ART are less absent than those not receiving treatment [28].

Estimates of PDL are higher for Zambia than for South Africa. It is possible that PDLs are affected by the time lost accessing healthcare, rather than inability to work because of sickness. As guidelines for both countries stipulate quarterly clinic visits for PLWH, the differences are likely explained by variations in travel and clinic waiting timings between the two countries. However, as the proportion of PLWH not on ART is only slightly higher in South Africa, it is unlikely that barriers to access can explain all differences. We could not find comparable empirical estimates of waiting times for the two countries; an evidence gap that requires further research. If PDLs were mainly explained by inefficiencies in accessing care, then they could possibly be reduced by supply-side interventions. Differentiated models of care policies, such as community pick-up points and adherence clubs, are being rolled out in both countries. They aim to shorten the time required to pick up drugs, and promise to remove or lower existing access barriers with possibly positive effects for PDLs [29].

This study has limitations. First, PDLs are based on self-reports and did not account for reduced productivity on working days, possibly underestimating productivity losses. Most previous studies have used employment records, but these are not available for informal sector workers and individuals not in the labour force. It is also difficult to measure reduced productivity while working. Second, we had no information on individuals’ clinical disease stage, and so stratified PDLs for PLWH by self-reported time on ART, which could have been affected by recall bias. This would not affect our overall estimates, but potentially those by treatment stage. However, mean CD4^+^ cell count at ART initiation has remained at about 152/μl in the past decade in sub-Saharan Africa [30]. Our results for Zambia suggest that after 2 years on ART, PDLs recover almost to those of individuals in earlier disease stages, a finding corroborated by previous studies on HIV-positive workers [12,13,25–27]. We also had to rely on self-reports of ART initiation amongst those self-reporting being HIV-positive, which may have resulted in some over-classification of individuals into the ‘not being on ART category.’ Third, we could not control for all covariates that may affect PDLs, for example, the presence of other working age individuals in the household, something not assessed in our sample. Moreover, women are overrepresented in our sample, which may bias our findings. However, we control for sex in all models and the predicted PDLs for men and women are very similar in Zambia, and not statistically different in South Africa. Finally, our data comes from communities in urban and periurban areas with comparably high HIV prevalence, and are therefore, not necessarily representative of other communities in the two countries.

We have calculated the days of work and home productivity lost to illness of all individuals irrespective of whether they were in the labour force, overcoming ethical issues that arise when comparing the benefits of interventions between individuals who are working and those who are not, even if they make positive contributions to society. These estimates could be used to calculate the opportunity costs of HIV in monetary terms, for example, by multiplying the estimates with gross domestic product (GDP) per capita or minimum wage rates. However, micro estimates of productivity, such as ours are incorrect estimates of future financial gains resulting from prevention or treatment interventions; they may underestimate or overestimate the aggregate productivity benefits from improved health [31]. Projection of the future macroeconomic impact requires more complex general equilibrium modelling, which considers additional factors, such as the degree to which infections are concentrated in hard-to-replace skilled workers, levels of unemployment, the impact of interventions on life expectancy, education, migration and changes in public and private savings or investments [7,31].

Our results provide estimates of the burden of the HIV epidemic resulting from lost work and home productivity in Zambia and South Africa. These will be a crucial input for modelling studies that aim to calculate the number of days lost to sickness that could be averted through programs of enhanced HIV prevention and treatment, and to comprehensively assess the economic benefit of such programs. We generated predictions of PDLs in various subgroups so that our findings are useful for a wide range of future studies. UNAIDS policies directed at achieving the ambitious 90–90–90 targets [32], are partly motivated by estimates of improved work productivity generated by simulation studies [8,9]. Our findings help to assess the validity of the assumptions on which these studies were based. For example, our results showed that HIV-negative workers do not have a null absenteeism rate (previous studies assumed that they do), and that labour productivity of persons on ART for three or more years is very similar to asymptomatic HIV-infected adults (previous studies assumed that it is substantially less) [8,9].

As part of the United Nations’ Sustainable Development Goals, the world has pledged to end the AIDS epidemic as a public health threat by 2030. To reach this ambitious goal, UNAIDS estimates that domestic and international investments in HIV programs in LMICs need to increase by about one-third, from an estimated US$ 19.1 billion in 2016 to US$ 26.2 billion until 2020 [33]. This represents a substantial allocation of resources that might otherwise be used for alternative worthwhile projects. At the country level, HIV interventions must compete against public investments into other interventions in the areas of health, education, infrastructure, housing, or agriculture. The benefits of these investments are commonly assessed on basis of their economic returns. It is difficult for policy makers to compare the benefits of the large investments needed to end the epidemic when their returns are only measured in terms of health outcomes, even if those are substantial. The findings from this study form an important contribution towards building a comprehensive and accurate investment case for HIV prevention and treatment interventions based upon their monetary benefits.

## Acknowledgements

We are grateful to all members of the HPTN 071 (PopART) Study Team and to the study participants and their communities for their contributions to this research. We are grateful for comments from Ronelle Burger (Stellenbosch University, South Africa), Sam Griffith (FHI 360, USA), Gesine Meyer-Rath (Wits University, Johannesburg, South Africa & Boston University, USA), and Andrew Mirelman (University of York, United Kingdom) for comments on earlier versions of this article.

Funding: HPTN 071 is sponsored by the National Institute of Allergy and Infectious Diseases (NIAID) under Cooperative Agreements UM1-AI068619, UM1-AI068617, and UM1-AI068613, with funding from PEPFAR. Additional funding is provided by 3ie with support from the Bill & Melinda Gates Foundation, as well as by NIAID, the National Institute on Drug Abuse (NIDA), and the National Institute of Mental Health (NIMH), all part of NIH. The content is solely the responsibility of the authors and does not necessarily represent the official views of the NIAID, NIMH, NIDA, PEPFAR, 3ie, or the Bill & Melinda Gates Foundation. K.H. was also partly funded by the National Institute for Health Research Health Protection Research Unit in Modelling Methodology at Imperial College London in partnership with Public Health England, and by the Centre funding from the UK Medical Research Council and Department for International Development, MRC Centre for Global Infectious Disease Analysis, reference MR/R015600/1by.

Contributors: R.T. and K.H. both conceived and designed the work. R.T. conceived and led on the statistical analysis and contributed to drafting and revising the article. K.H. took the lead on writing and revising the article and contributed to analysis and interpretation of the data. R.F. and K.B. contributed to the analysis of the data and revising of the article. All other authors contributed to the conception or design of HPTN071 (PopART), interpretation of data for the work, acquisition of the data and revision of the article.

### Conflicts of interest

K.H. and R.T. received personal fees from the international Decision Support Initiative for work unrelated to this study. K.H. also received personal fees from The Global Fund for work unrelated to this study.

## Supplementary Material

Supplemental Digital Content
