# Supplementary material for: Work and home productivity of people living with HIV in Zambia and South Africa
Source: AIDS. 2019 Apr 4;33(6):1063–71. doi: 10.1097/QAD.0000000000002160 (PMC6467557; doi:10.1097/QAD.0000000000002160)
Supplement: Supplemental Digital Content [file aids-33-1063-s001.docx]

**Work and home productivity of people living with HIV in Zambia and South Africa**

Supplementary Material

**Table A1: Previous studies**

| Author | Pub year | Data year | Modelling | Setting | Sample (HIV+) | HIV status | Comparison group | Sample characteristics | Excess sickness days (over 3 months) |
| --- | --- | --- | --- | --- | --- | --- | --- | --- | --- |
| Leigh | 1997 | 1990-1992 | Econometric 2-part model | Five outpatient practices, USA | 884 (541) | HIV+ not AIDS; HIV+ AIDS | HIV- | Employed patients | 5∙1 days (AIDS patients)  No difference (HIV+ patients not AIDS) |
| Fox | 2004 | 1997-2002 | Random effects panel data model | Tea plantation, Kenya | 271 (54) | HIV+ who had died | Unknown HIV status | Male and female employees | 2∙3 – 2∙75 (sick leave);  1∙6 -2∙075 (annual leave);  2∙95 - 4∙975 (casual leave, all final year of life) |
| Rosen | 2007 | 2003-2005 | Linear regression | Zambia Wildlife Authority | 87 (11) | HIV+ who had died | Unknown HIV status | Male employees | 25∙7 days on average (patrol days missed)  33∙7 in final year of life |
| Larson | 2008 | 2004-2006 | Difference-in-difference analysis | Tea plantation, Kenya | 2051 (59) | HIV+ 2 years before and 1 year after ART initiation | Unknown HIV status | Employees | 4∙2 days (not on ART)  5∙1 days (on ART) |
| Larson | 2009 | 2004-2006 | Nearest neighbour matching | Tea plantation, Kenya | 2485 (97) | HIV+ 2 years before and 1 year after ART initiation | Unknown HIV status | Male and female employees | 5∙52 (males pre-ART)  No difference (males on ART 7m+)  9∙96 to 15∙63 days (females pre-ART)  11∙6 to 31∙7 days (females on ART 7m+) |
| Sonnenberg | 2010 | 1992-2002 | Logistic regression | 4 gold mines, South Africa | 6,562 (1703) | HIV+ | HIV- | Male employees | 3 days  18 days (final year of life) |
| Habyarimana | 2010 | 1998-2006 | Fixed effects panel data model | Mining company, Botswana | 7,661 (721) | HIV+ recently initiated on ART; HIV+ enrolled but not on ART | HIV- and HIV+ not enrolled | Employees | 5 days (HIV+ in year before initiation)  No difference (HIV+ enrolled but not on ART) |
| Guariguata | 2012 | 2009-2010 | Negative binomial regressions | 42 companies in 7 industries, Namibia | 7,666 (694) | HIV+ | HIV- | Employees | 1∙55 Incidence rate ratio for HIV+ (It was not possible to quantify result in days based on data provided in paper) |
| Larson | 2013 | 2004-2007 | Nearest neighbour matching | 2 tea plantations, Kenya | 1185 (237) | HIV+ 2 years before and 2 years after ART initiation | Unknown HIV status | Employees | 3∙3 (males not on ART)  6 (males on ART)  No differences (females not on ART)  5 (females on ART) |

**A2: Details on community selection in the trial**

In the study, community is defined as the catchment population of a local health unit, through which antiretroviral treatment (ART) for HIV is delivered. The 21 communities in Zambia and South Africa were selected for their high HIV prevalence and incidence. Adult HIV prevalence ranges from about 11% to 25%, with an average of around 15% to 17% in both countries. The communities were selected taking into consideration whether they were geographically distinct areas, there were no major HIV preventions studies planned or ongoing, were large enough in terms of population size to allow measurement of population level HIV incidence, and the communities were willing to participate in the trial. The figure below shows the study sites in Zambia and South Africa. The following table presents characteristics of the study communities.

**
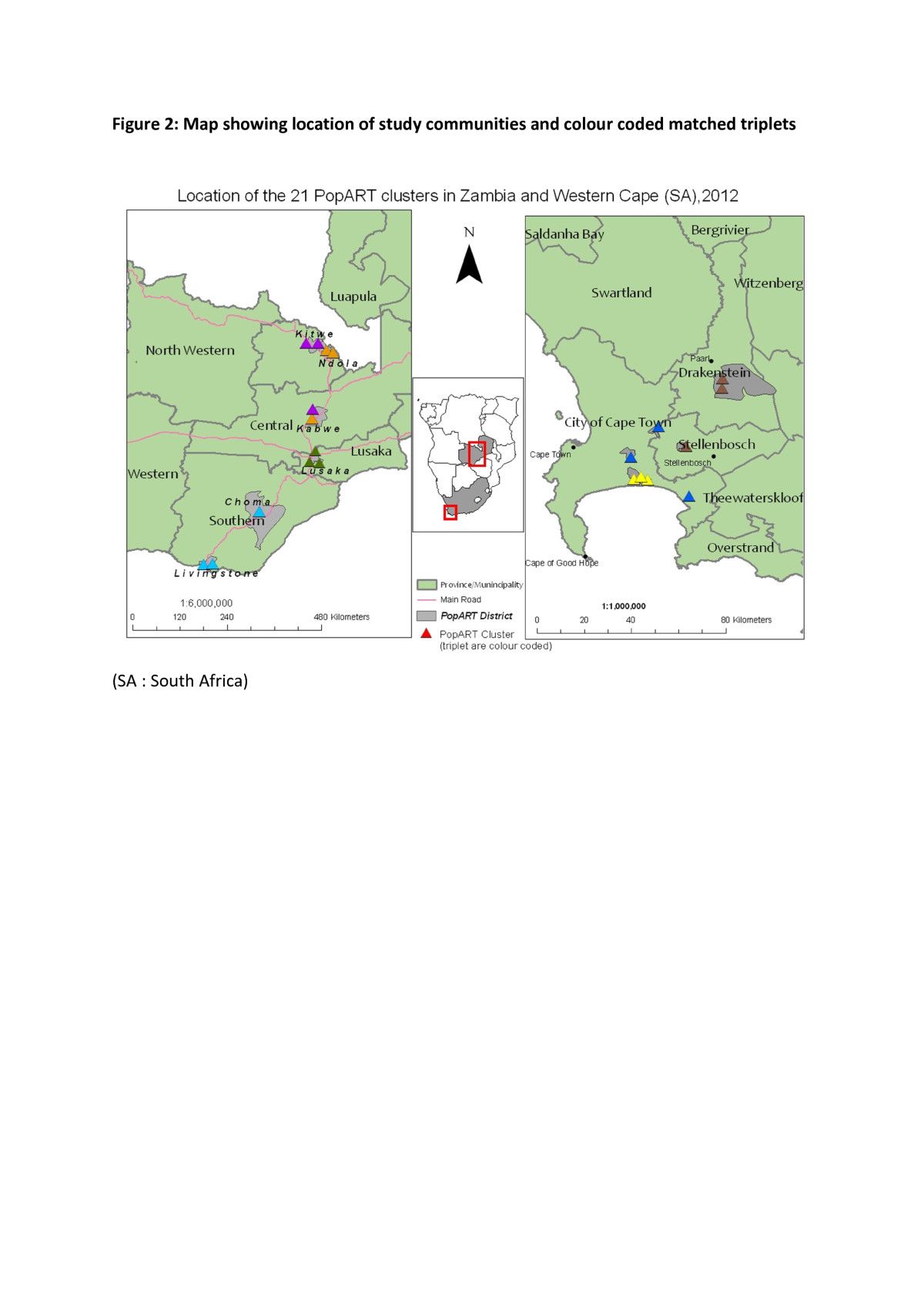
**

**Characteristics of study communities taken from Hayes et al [1]**

| **Country** | **Community number** | **Adult HIV prevalence (%) ^1^** | **HIV-infected on ART (%) ^2^** | **Population size ^3^** |
| --- | --- | --- | --- | --- |
| Zambia | 1 | 16 | 23 | 42,898 |
|  | 2 | 13 | 29 | 33,297 |
|  | 3 | 17 | 15 | 38,081 |
|  | 4 | 19 | 30 | 60,222 |
|  | 5 | 17 | 18 | 45,234 |
|  | 6 | 19 | 32 | 34,623 |
|  | 7 | 16 | 13 | 129,221 |
|  | 8 | 15 | 22 | 166,251 |
|  | 9 | 16 | 25 | 124,284 |
|  | 10 | 25 | 24 | 31,629 |
|  | 11 | 18 | 27 | 55,011 |
|  | 12 | 16 | 38 | 41,615 |
| South Africa | 13 | 19 | 35 | 34,096 |
|  | 14 | 19 | 35 | 21,386 |
|  | 15 | 19 | 35 | 38,059 |
|  | 16 | 15 | 37 | 72,544 |
|  | 17 | 18 | 28 | 37,084 |
|  | 18 | 14 | 36 | 44,821 |
|  | 19 | 11 | 25 | 36,009 |
|  | 20 | 11 | 25 | 82,953 |
|  | 21 | 12 | 18 | 45,067 |
| Notes:  Source: Information in the table replicated from Hayes et al [1]  The identities of the communities are concealed because the PopART trial is still ongoing.  ^1^Estimated from ZAMSTAR 2010 TB/HIV prevalence survey, for all Zambian communities, with age-standardisation to the age structure of prevalence survey participants and assuming 50% of the adult population are men. For Western Cape communities, source of HIV prevalence data varies by triplet. For Triplet 5, community 13 was included in the ZAMSTAR trial and the ZAMSTAR 2010 TB/HIV prevalence survey data are used, as for Zambia. HIV prevalence is then assumed to be the same in communities 14 and 15. For communities 16, 17, 19, 20, and 21, sub-district level data on antenatal clinic (ANC) prevalence were used, with the assumption that adult HIV prevalence is 80% of the ANC prevalence value. Community 18 was included in the ZAMSTAR trial and the ZAMSTAR 2010 TB/HIV prevalence survey data are used.  ^2^Estimated from ZAMSTAR 2010 TB/HIV prevalence survey data, for all Zambian communities. The number of HIV-positive adults among prevalence survey participants was estimated, separately for men and women, as the age-standardised HIV prevalence multiplied by the number of survey participants. The proportion of HIV-positive individuals on ART was then calculated as (number self-reported on ART)/(estimated number of HIV-positive survey participants), and assuming that 50% of the adult population are men. For Western Cape communities, data were used from October 2012 on (a) the number of individuals aged >15 years old on ART – measured either at community or sub-district level, (b) population size among individuals >15 years old – measured using census data either at community or sub-district level, and (c) HIV prevalence estimates. The number of HIV-positive individuals aged >15 years old was estimated as HIV prevalence × community (or sub-district) population size. The proportion of HIV-positive individuals on ART was then calculated as (number of individuals >15 years old on ART)/(estimated number of HIV-positive individuals aged >15 years old).  ^3^Population size – for Zambia, based on 2001 census data; for Western Cape, based on 2011 census data. | | | | |

**A3: Details on blood sampling and HIV testing**

Blood samples were analysed in-country using a single 4th generation assay (Architect HIV Ag/Ab Combo Assay, Abbott Diagnostics, Delkenheim Germany). Further testing was performed at the HIV Prevention Trials Network (HPTN) Laboratory Center (Baltimore, MD, USA). Samples that had reactive results in-country were tested with a second 4th generation assay (GS HIV Combo Assay, Bio-Rad Laboratories, Redmond, Wa). For quality assurance, 10% of the samples that had non-reactive results in-country were tested again using the Architect HIV Ag/Ab Combo Assay. Samples with discrepant/discordant test results were tested with additional assays to determine HIV status.

**A4: Selection of survey participants**

In each of the 21 trial communities, a random sample of households was selected and visited by field staff who enumerated all adults aged 18–44 years. From this list, one adult from each household was randomly selected and provided informed consent to participate in the population cohort. If a selected individual declined to participate or was found ineligible, then the fieldworkers moved on to the next household. No replacement individual was selected from the same household. If the selected individual was not available at the time of enumeration, the contact information of the selected potential participant was obtained and research assistants contacted the participant to find out when they were most likely be home and able to complete the interview. Three attempts were made to contact the potential participant in person. Eligibility criteria included: being between ages 18-44, able and willing to provide informed consent; residing within catchment area of a designated local health unit and intending to remain so for the next three years and residing in a randomly selected household. Exclusion criteria included current or planned enrolment in another HIV treatment, prevention, or PrEP study and current, planned or prior enrolment in an HIV vaccine study.

**Table A5: Distribution of self-reported productive days lost over three months in a random sample of adults 18-44 years of age in 21 communities in Zambia and South Africa**

| PDLs | Zambia (%)  N=18823 | South Africa (%)  N=17527 |
| --- | --- | --- |
| 0 | 86∙01 | 95∙86 |
| 1 | 1∙16 | 0∙74 |
| 2 | 1∙75 | 0∙92 |
| 3 | 2∙42 | 0∙74 |
| 4 | 1∙22 | 0∙26 |
| 5 | 0∙84 | 0∙27 |
| 6 | 0∙22 | 0∙07 |
| 7 | 3∙28 | 0∙25 |
| 8 | 0∙22 | 0∙19 |
| 9 | 0∙03 | 0∙01 |
| 10 PDLs or more | 2∙85 | 0∙69 |

**Table A6: Model fit comparison between Poisson and Negative Binomial**

|  | Zambia | | | | | South Africa | | | | |
| --- | --- | --- | --- | --- | --- | --- | --- | --- | --- | --- |
|  | **Actual** | **Predicted - Model 1a** | | **Predicted- Model 2a** | | **Actual** | **Predicted-Model 2a** | | **Predicted-Model 2b** | |
| Count |  | **Poisson** | **Negative binomial** | **Poisson** | **Negative binomial** |  | **Poisson** | **Negative binomial** | **Poisson** | **Negative binomial** |
| 0 | 0∙86 | 0∙35 | 0∙85 | 0∙35 | 0∙85 | 0∙96 | 0∙77 | 0∙96 | 0∙78 | 0∙96 |
| 1 | 0∙01 | 0∙31 | 0∙04 | 0∙31 | 0∙04 | 0∙01 | 0∙19 | 0∙01 | 0∙18 | 0∙01 |
| 2 | 0∙02 | 0∙18 | 0∙02 | 0∙18 | 0∙02 | 0∙01 | 0∙04 | 0∙01 | 0∙03 | 0∙01 |
| 3 | 0∙03 | 0∙09 | 0∙01 | 0∙09 | 0∙01 | 0∙01 | 0∙01 | 0∙00 | 0∙01 | 0∙00 |
| 4 | 0∙01 | 0∙04 | 0∙01 | 0∙04 | 0∙01 | 0∙00 | 0∙00 | 0∙00 | 0∙00 | 0∙00 |
| 5 | 0∙01 | 0∙02 | 0∙01 | 0∙02 | 0∙01 | 0∙00 | 0∙00 | 0∙00 | 0∙00 | 0∙00 |
| 6 | 0∙00 | 0∙01 | 0∙01 | 0∙01 | 0∙01 | 0∙00 | 0∙00 | 0∙00 | 0∙00 | 0∙00 |
| 7 | 0∙03 | 0∙00 | 0∙01 | 0∙00 | 0∙01 | 0∙00 | 0∙00 | 0∙00 | 0∙00 | 0∙00 |
| 8 | 0∙00 | 0∙00 | 0∙00 | 0∙00 | 0∙00 | 0∙00 | 0∙00 | 0∙00 | 0∙00 | 0∙00 |
| 9 | 0∙00 | 0∙00 | 0∙00 | 0∙00 | 0∙00 | 0∙00 | 0∙00 | 0∙00 | 0∙00 | 0∙00 |
| 10 | 0∙00 | 0∙00 | 0∙00 | 0∙00 | 0∙00 | 0∙00 | 0∙00 | 0∙00 | 0∙00 | 0∙00 |
| 11 | 0∙00 | 0∙00 | 0∙00 | 0∙00 | 0∙00 | 0∙00 | 0∙00 | 0∙00 | 0∙00 | 0∙00 |
| 12 | 0∙00 | 0∙00 | 0∙00 | 0∙00 | 0∙00 | 0∙00 | 0∙00 | 0∙00 | 0∙00 | 0∙00 |
| 13 | 0∙00 | 0∙00 | 0∙00 | 0∙00 | 0∙00 | 0∙00 | 0∙00 | 0∙00 | 0∙00 | 0∙00 |
| 14 | 0∙01 | 0∙00 | 0∙00 | 0∙00 | 0∙00 | 0∙00 | 0∙00 | 0∙00 | 0∙00 | 0∙00 |
| 15 | 0∙00 | 0∙00 | 0∙00 | 0∙00 | 0∙00 | 0∙00 | 0∙00 | 0∙00 | 0∙00 | 0∙00 |
| 16 | 0∙00 | 0∙00 | 0∙00 | 0∙00 | 0∙00 | 0∙00 | 0∙00 | 0∙00 | 0∙00 | 0∙00 |
| 17 | 0∙00 | 0∙00 | 0∙00 | 0∙00 | 0∙00 | 0∙00 | 0∙00 | 0∙00 | 0∙00 | 0∙00 |
| 18 | 0∙00 | 0∙00 | 0∙00 | 0∙00 | 0∙00 | 0∙00 | 0∙00 | 0∙00 | 0∙00 | 0∙00 |
| 19 | 0∙00 | 0∙00 | 0∙00 | 0∙00 | 0∙00 | 0∙00 | 0∙00 | 0∙00 | 0∙00 | 0∙00 |
| 20 | 0∙00 | 0∙00 | 0∙00 | 0∙00 | 0∙00 | 0∙00 | 0∙00 | 0∙00 | 0∙00 | 0∙00 |

Note: Comparison of the probability masses at low counts show very small differences between actual and predicted probabilities for the negative binomial models compared to the Poisson model.

**References**

1. Hayes R, Ayles H, Beyers N, Sabapathy K, Floyd S, Shanaube K, et al. **HPTN 071 (PopART): rationale and design of a cluster-randomised trial of the population impact of an HIV combination prevention intervention including universal testing and treatment - a study protocol for a cluster randomised trial**. *Trials* 2014; 15:57.
